# Central deficiency of IL-6Ra in mice impairs glucose-stimulated insulin secretion

**DOI:** 10.1016/j.molmet.2022.101488

**Published:** 2022-04-22

**Authors:** Alison D. McNeilly, Adonis Yianakas, Jennifer G. Gallagher, Jamie Tarlton, Michael LJ. Ashford, Rory J. McCrimmon

**Affiliations:** Division of Systems Medicine, School of Medicine, University of Dundee, Ninewells Hospital and Medical School, Dundee, DD1 9SY, UK

**Keywords:** Interleukin-6, Food intake, Energy expenditure, Insulin secretion, Autonomic output

## Abstract

**Objective:**

IL-6 is an important contributor to glucose and energy homeostasis through changes in whole-body glucose disposal, insulin sensitivity, food intake and energy expenditure. However, the relative contributions of peripheral versus central IL-6 signaling to these metabolic actions are presently unclear. A conditional mouse model with reduced brain IL-6Ra expression was used to explore how blunted central IL-6 signaling alters metabolic status in lean and obese mice.

**Methods:**

Transgenic mice with reduced levels of central IL-6 receptor alpha (IL-6Ra) (*IL-6Ra KD* mice) and Nestin Cre controls (*Cre*^*+/-*^ mice) were fed standard chow or high-fat diet for 20 weeks. Obese and lean mouse cohorts underwent metabolic phenotyping with various measures of energy and glucose homeostasis determined. Glucose-stimulated insulin secretion was assessed *in vivo* and *ex vivo* in both mouse groups.

**Results:**

*IL-6Ra KD* mice exhibited altered body fat mass, liver steatosis, plasma insulin, IL-6 and NEFA levels versus *Cre*^*+/-*^ mice in a diet-dependent manner. *IL-6Ra KD* mice had increased food intake, higher RER, decreased energy expenditure with diminished cold tolerance compared to *Cre*^*+/-*^ controls. Standard chow-fed *IL-6Ra KD* mice displayed reduced plasma insulin and glucose-stimulated insulin secretion with impaired glucose disposal and unchanged insulin sensitivity. Isolated pancreatic islets from standard chow-fed *IL-6Ra KD* mice showed comparable morphology and glucose-stimulated insulin secretion to *Cre*^*+/-*^ controls. The diminished *in vivo* insulin secretion exhibited by *IL-6Ra KD* mice was recovered by blockade of autonomic ganglia.

**Conclusions:**

This study shows that central IL-6Ra signaling contributes to glucose and energy control mechanisms by regulating food intake, energy expenditure, fuel flexibility and insulin secretion. A plausible mechanism linking central IL-6Ra signaling and pancreatic insulin secretion is through the modulation of autonomic output activity. Thus, brain IL-6 signaling may contribute to the central adaptive mechanisms engaged in response to metabolic stress.

## Highlights

• Lower brain IL-6 signaling increases food intake and decreases energy expenditure.
• Reduced metabolic flexibility associated with lower central IL-6 signaling.
• Blunted central IL-6 signaling inhibits glucose-stimulated insulin secretion.
• Diminished brain IL-6 signaling alters autonomic drive to pancreatic beta cells.

## 1 Introduction

Interleukin (IL)-6 has been identified as an important contributor to energy and glucose homeostasis. Plasma IL-6 levels correlate positively with increased fat mass in rodents and humans [[1], [2], [3], [4]], with levels reduced on weight loss [5]. In contrast, the cerebrospinal level of IL-6 in humans is reported to be negatively correlated with obesity [6], and central IL-6 expression in obese rodents is reduced [7]. Consequently, it is plausible that the function of central IL-6 differs from peripheral IL-6. Peripheral administration of IL-6 in mice induces insulin resistance [8,9], and peripheral IL-6 antibody neutralisation of IL-6 in a transgenic diabetes mouse model improves hepatic insulin sensitivity [10]. Central IL-6 administration results in decreased food intake, reduced fat mass and increased energy expenditure in rodents [[11], [12], [13]], with IL-6 antibody neutralisation of central IL-6 preventing the reduction in food intake [14]. Interestingly, central IL-6 produced only a moderate improvement of glucose disposal in lean mice, whereas, in high-fat diet-fed obese mice, central IL-6 significantly improved glucose disposal and insulin sensitivity [14].

In addition, the presence of central IL-6 may be required for the full response of leptin, amylin and GLP-1 to cause inhibition of food intake and reduction of body weight [7,[15], [16], [17]]. In support of a predominating central role, whole body IL-6 null mice exhibit increased body weight, and fat mass with insulin resistance [12,18] and transgenic mice overexpressing human IL-6 in the brain and lung remain lean, are more insulin sensitive, and are protected from diet-induced obesity, potentially by increased leptin action [19]. Thus, the consensus view is that many metabolic actions of IL-6 are generated centrally, with the hypothalamus considered the predominant site [14,20,21]. However, recently the lateral parabrachial nucleus (PBN) has been implicated in mediating the actions of IL-6 on food intake and energy expenditure [7].

IL-6 engenders signaling by binding to IL-6 receptor alpha (IL-6Ra), which requires association with the transmembrane protein gp130 to produce a functional signaling complex. Additionally, a soluble form of IL-6Ra (sIL-6R) exists, produced by enzymatic cleavage in rodents, and enables IL-6 signaling in cells devoid of IL-6Ra. The binding of IL-6 to sIL-6R in plasma and CSF permits this complex to associate with gp130, which is ubiquitously expressed, and elicit IL-6-dependent downstream signaling [22]. This alternative IL-6 pathway is termed trans-signaling to differentiate it from the classical IL-6-IL-6Ra pathway.

IL-6 signaling in both neurons and astrocytes plays important roles in glucose and energy homeostasis. In mice fed a high-fat diet, with IL-6 deleted from neurons or astrocytes only, neuron IL-6 null mice display resistance to obesity with reduced levels of insulin resistance and loss of glucose homeostasis. In contrast, astrocyte IL-6 null mice are more susceptible to weight gain [23,24]. However, it is likely that trans-signaling, rather than classical IL-6R signaling, is responsible for this central modulation of energy and glucose homeostasis [14,25]. Indeed, reduced food intake following central delivery of IL-6 is maintained in mice deficient in hypothalamic or forebrain IL-6Ra, whereas preventing central IL-6 trans-signaling blocks central IL-6 mediated food intake reduction [14].

Thus, the mechanisms responsible for the beneficial actions of central IL-6 mediated signaling on whole-body energy, and glucose homeostasis are still unclear. Consequently, we utilised a conditional mouse model with diminished IL-6Ra brain expression to examine how blunted central IL-6 signaling affects metabolic status in lean and obese mice.

## 2 Material and methods

### 2.1 Animal ethics and husbandry

Homozygous *IL-6Ra*^*flx/flx*^ (B6 (SJL)-*Il6ra*^*tm.1.1Drew*^*/J*; Jackson Laboratories) mice were crossed with heterozygous Nestin *Cre*^*+/-*^ mice (kindly provided by Simon Arthur, University of Dundee) to generate *IL-6Ra*^*+/-*^ knock-down (*IL-6Ra KD*) mice and heterozygous *IL-6Ra*^*flx+/Cre-*^ littermate controls (*WT*^*flx*^). Nestin-*Cre*^*+/-*^ (*Cre*^*+/-*^) mice were used as primary controls in this study as Nestin-*Cre* mice exhibit a metabolic phenotype slightly different from wild-type mice [[26], [27], [28]]. Mice were housed in groups of 2–5, depending on litter size at 20 °C - 21 °C and fed *ad libitum* with access to water on a 12:12 h light: dark schedule. All animal procedures were approved by the University of Dundee Ethical Review Process and performed in accordance with UK Home Office regulations (under the auspices of Project License PIL60/4285). All experiments were performed on adult male mice as female mice have been reported to be unresponsive to HFD-mediated reduction in IL-6 central expression [7].

### 2.2 Experimental groups and metabolic analyses

Male *IL-6Ra KD* and *Cre*^*+/-*^ mice (12–14 weeks of age) were randomly assigned to receive either standard rodent chow (SC; RM1-SDS diets, UK: by energy 7.4% crude fat, 17.5% crude protein, 75.1% carbohydrate) or a high-fat diet (HF; (SDS 824053), by energy 45% crude fat (lard), 20% crude protein, 35% carbohydrate) (*n* = 10–12 per group) and remained on this diet for the duration of the experiment (20 weeks). Bodyweight was measured weekly, and body composition was assessed by EchoMRI at weeks 7 and 14. Glucose sensitivity was assessed by oral glucose tolerance test (oGTT, set dose of 100ul of 50% glucose solution) and insulin sensitivity by insulin tolerance test (ITT; 0.75mU/g insulin i.p. body weight) following a 5hr fast at weeks 10 and 15, respectively. Oral glucose (set dose of 100ul of 50% glucose solution) - stimulated insulin secretion (oGSIS) test was performed at week 19 with blood samples collected for the measurement of insulin at 0, 3, 15 and 30 min. Insulin levels were measured by ELISA (Crystal Chem® Ultrasensitive insulin ELISA). In all circumstances, blood glucose was measured from the tail vein using a hand-held glucose meter (Accuread®). Whole-body metabolism was measured using the CLAMS (Comprehensive Lab Animal Monitoring System, Columbus©) system at the end of the study (week 20). Respiratory exchange ratio (RER), food intake, activity and energy expenditure were assessed over 72 h with measurements taken every 12 min. The first 24 h when animals were habituated to the novel monitoring chambers were excluded from the analysis. Plasma leptin and IL-6 levels were measured in trunk blood by ELISA (Quantikine, R&D Systems), and triglycerides and non-esterified fatty acids (NEFA) fluorometrically using commercially available kits (Abcam).

### 2.3 RNA extraction and PCR

Total RNA was extracted from brain regions (hippocampus, frontal cortex, cerebellum and hypothalamus), pancreas and liver from male *IL-6Ra KD* and *Cre*^*+/-*^ mice using TRIzol® reagent (Invitrogen). Reverse transcription was performed with 1 ng RNA using SuperScript® III First-Strand Synthesis System for RT (Invitrogen). Real-time PCR was performed using Taqman gene expression assays for *IL-6ra* (Mm01211445_m1), *Socs3* (Mm00545913_s1) and *Igfbp1* (Mm00515154_m1). All samples were performed in triplicate and normalised to Cyclophilin A (*Ppia;* Mm02342430_g1) or actin (Mm02619580_g1). Values are expressed as a fold-change relative to *Cre*^*+/-*^.

### 2.4 Liver histology

Hepatic Lipid accumulation was assessed in frozen sections (30 μm) mounted on to charged slides. Frozen sections were allowed to come to room temperature, rinsed briefly in nuclease-free H_2_0 then immersed in propan-2-ol (60%) for 30s. Subsequently, sections were submerged in Oil red O (10 min) then washed thoroughly in nuclease-free H_2_0 to remove excess dye. Finally, sections were immersed in hematoxylin (1 min), rinsed in nuclease-free H_2_0 then briefly immersed in ammonia (1%; 30s) before mounting.

### 2.5 Temperature challenge

To assess the thermoregulatory response to a cold temperature challenge, SC-fed *Cre*^*+/-*^ and *IL-6Ra KD* mice were individually housed in the absence of bedding and placed at 4 °C for 6 h. Core body temperature was measured using a rectal probe at 30 min intervals. The change (delta) in body temperature was calculated as the difference between core temperature at 0 and 6 h.

### 2.6 Islet isolation and ex-vivo GSIS

Islets were isolated from SC- and HF-fed male *Cre*^*+/-*^ and *IL-6Ra KD* mice at week 18 of the dietary intervention as described previously [29]. Briefly, islets were incubated in a 24 well plate (10 islets per well) overnight in 500 μl of RPMI-1640 containing 10% FBS, 1% Penicillin/Streptomycin and 2 mmol/l l-Glutamine at 37 °C. After 24 h in culture, islets were transferred to 500 μl Krebs-HEPES-bicarbonate (KHB) solution (in mmol/l: 130 NaCl, 3.6 KCl, 1.5 CaCl_2_, 0.5 MgSO_4_, 0.5 KH_2_PO_4_, 2 NaHCO_3_, 10 HEPES, and 0.1% (wt/vol) BSA, pH 7.4) containing 2.8 mmol/l glucose for 1 h at 37 °C prior to measurement of glucose-stimulated insulin secretion, as described [30]. Islets (10 per well) were incubated at a glucose concentration of 2.8 or 16.5 mmol/l or KCl (30 mmol/l) for 30 min in 250 μl KHB. After the final time point, total islet content was extracted into 50 μl of acid-ethanol-Triton solution [1.5% (vol/vol) HCl, 75% (vol/vol) ethanol, 0.1% (vol/vol) Triton X-100]. The secreted (0, 3, 15 and 30 min) insulin and the total islet insulin content were measured using a multiplex detection ELISA (Bio-plex®, Biorad). In a separate cohort of animals (n = 4 per group), animals were sacrificed, the pancreas isolated and fixed in 4% PFA for subsequent immunohistochemical analysis. Subsequently, paraffin-embedded sections (5 μm) were stained with primary antibodies for insulin (Anti-Insulin; Abcam ab7842; 1:100) or glucagon (Anti-Glucagon; Sigma Aldrich SAB451137, 1:100) at 4 °C overnight. Sections were washed in PBST and incubated with appropriate secondary antibodies (Cy™3; Jackson ImmunoResearch® 1:250) and Alexa Fluor 488 (Life Technologies 1:500) for beta and alpha cells. Images were acquired using a confocal laser scanning microscope (Leica® TCS SP5 II) at x40 magnification. Quantification was performed using Volocity® cellular imaging and analysis software (Perkin Elmer) by Dr Alison Milne, Light Microscopy Facility, School of Medicine Dundee.

### 2.7 Hexamethonium blockade

Mice (n = 4 per group) were fasted for 4 h and injected with the non-depolarising autonomic ganglionic blocker hexamethonium bromide (Sigma–Aldrich), dissolved in saline (30 mg/kg i.p.). After 15 min, an oGSIS test was performed, as described in *2.2*. Blood glucose and blood samples were collected from the tail vein at 0, 3, 15 and 30 min, and insulin levels were measured by multiplex detection ELISA (Milliplex©, Merck).

### 2.8 IL-6 and leptin stimulation

Mice (n = 4 per group) were fasted for 5 h before stimulation with either IL-6 (50 μg/kg i.p.), Leptin (3 mg/kg i.p.) or saline (Control). After 30 min, the mice were killed by cervical dislocation and tissue (liver, skeletal muscle, white adipose tissue (WAT) and hypothalamus) isolated and snap-frozen in liquid nitrogen for subsequent biochemical analysis.

### 2.9 Phospho-STAT3 analysis

The levels of phospho-STAT3 (p-STAT3 (Y^705^)) within the hypothalamus, liver, skeletal muscle and WAT were measured by western blot analysis. Briefly, tissues were homogenised in lysis buffer and protein isolation, content, immunoblotting and analysis were performed as described [31]. Primary antibodies used were anti-phospho-STAT3 (Tyr^705^, 1:1000 dilution) and anti-GAPDH (1:2000 dilution, both Cell Signaling Technologies).

### 2.10 Ex-vivo hypothalamic brain slicing

Male *IL-6Ra KD* and *Cre*^*+/-*^ mice were maintained as described above. Animals were fasted for 1 h prior to experiments and slices prepared as described previously [31]. In brief, horizontal 400-μm coronal brain slices were prepared using a Vibratome (Intracel, Royston, UK) and basal-medial hypothalamic (BMH) wedges were cut and incubated in aCSF containing glucose (4.5 mmol/l) ± IL-6 (20 ng/ml) for 30 min. Following treatment, slices were homogenised in lysis buffer, and western blots were performed for p-STAT3 (Y705) and GAPDH as described in *2.7*.

### 2.11 Statistical analysis

All results are expressed as the mean ± SEM. Statistical analyses were performed using SPSS (version 21; SPSS) or GraphPad Prism (version 6). Datasets with more than two groups were analysed by one-way ANOVA or repeated-measures ANOVA followed by post hoc testing (Bonferroni test) to localise significance. All data were tested for normality using the Shapiro–Wilk normality test. All data, apart from densitometry analysis of immunoblots and relative body composition and mRNA expression, passed this test (*P* > 0.05). Consequently, these data were analysed for two or multiple groups using Mann–Whitney and Kruskal–Wallis tests, respectively.

## 3 Results

### 3.1 Generation and validation of IL-6Ra KD mice

**Figure 1:**
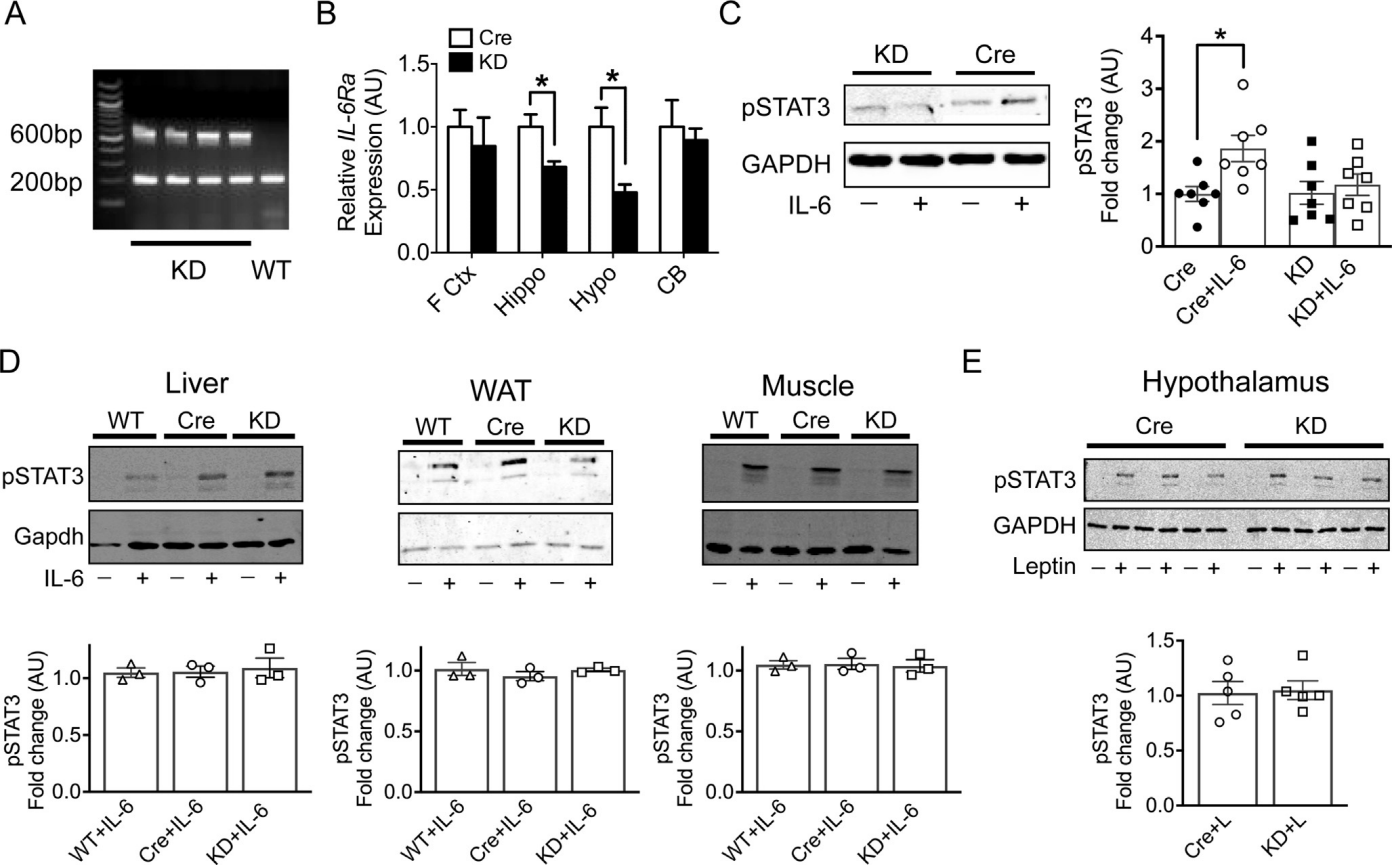
**Central knockdown of*****IL-6Ra*****expression.** A. Targeted deletion of exons 4–6 of the IL-6Ra gene by recombination of the floxed *IL-6Ra* allele detected by PCR (600 bp band). B. Quantification of IL-6Ra receptor mRNA expression in the frontal cortex (F Ctx), hippocampus (Hippo), hypothalamus (Hypo) and cerebellum (CB) in *IL-6Ra KD* (KD; *n* = 6) and *Cre*^*+/-*^ (Cre; *n* = 7) control mice. C. Representative immunoblots of IL-6 stimulated phosphorylation of STAT3 (pSTAT3) from basal-medial hypothalamic (BMH) slice wedges of *IL-6Ra KD* (*n* = 7) and *Cre*^*+/-*^ (*n* = 7) mice. The graph shows normalised means ± SEM of immunoblots for BMH slice wedges of the indicated genotype. D. Representative immunoblots of levels of pSTAT3 in the liver, white adipose tissue and skeletal muscle isolated from wild type (WT), *Cre*^*+/-*^ and *IL-6Ra KD* mice, stimulated with saline or IL-6 (50 μg/kg i.p.). Graphs denote fold change in p-STAT3 levels by IL-6 from tissues (*n* = 3/group) of the indicated genotypes. E. Representative immunoblots of levels of pSTAT3 in the hypothalamus of *Cre*^*+/-*^ control (Cre) and *IL-6Ra KD* (KD) mice, stimulated with leptin (3 mg/kg i.p.) or saline. Graph denotes fold change in p-STAT3 by leptin (*n* = 5/group). Results represent mean values ± SEM. Data were analysed by Kruskal–Wallis followed by Dunn's post-hoc test. ∗*P* < 0.05.

*IL-6Ra* mice have targeted deletion of exons 4–6 of IL-6Ra, a region that includes the cytokine-binding domain, as previously described [32]. Recombination was verified by PCR analysis of ear notches from *IL-6Ra* mice and *WT*^*flx*^ controls (Figure 1A). The expression of *Cre* and, therefore, reduced expression of IL-6Ra was confirmed by the presence of a band at 600bp. The 200bp band acts as a positive internal control. To determine the level of IL-6Ra receptor knock-down, mRNA abundance of *IL-6Ra* was assessed and compared with *Cre*^*+/-*^ controls in the hippocampus, hypothalamus, cerebellum and frontal cortex (Figure 1B). Receptor expression was significantly reduced in the hippocampus (32% reduction; *p* < 0.05, *IL-6Ra* vs *Cre*^*+/-*^) and hypothalamus (45% reduction; *p* < 0.05, *IL-6Ra* vs *Cre*^*+/-*^). There was no reduction in *IL-6Ra* mRNA abundance in either the cerebellum (*p* = 0.56) or frontal cortex (*p* = 0.68), suggesting a significant degree of region-specific transgene transcriptional repression. Thus, this *IL-6Ra* mouse line is best described as a limbic IL-6Ra knock-down (*IL-6Ra KD*) model. To assess the functional consequence of a reduction in *IL-6Ra* expression in the hypothalamus, basal-medial hypothalamic (BMH) slice wedges [31,33] were incubated in the presence or absence of IL-6 (20 ng/ml) for 30 min and the levels of p-STAT3 measured by western blot. Treatment with IL-6 increased BMH p-STAT3 expression in slices from *Cre*^*+/-*^ control mice (Figure 1C; *p* < 0.05). In contrast, there was no change in BMH p-STAT3 levels in *IL-6Ra KD* mice (*p* = 0.61), demonstrating that successful knock-down of *IL6Ra* in this region resulted in blunted IL-6-mediated signaling. To determine whether the *Cre* transgene expression had a significant peripheral off-target effect resulting in reduced *IL-6Ra* expression in other Nestin expressing tissues [28], *Cre*^*+/-*^, *WT*^*flx*^, and *IL-6Ra KD* mice were fasted for 5 h, injected with IL-6 (50 mg/kg i.p.) and p-STAT3 expression assessed in the liver, skeletal muscle, and WAT by western blot analysis (Figure 1D). In all peripheral tissues examined, IL-6 stimulated an increase in p-STAT3, indicating the presence of functional IL-6-mediated signaling in these tissues. Furthermore, administration of leptin (3 mg/kg i.p.) resulted in an equivalent robust increase in hypothalamic p-STAT3 levels in *Cre*^*+/-*^ and *IL-6Ra KD mice* (Figure 1E), indicating no reduction in leptin efficacy by IL-6Ra knock-down in this region. These findings confirm that IL-6 mediated signaling, as monitored by p-STAT3 levels, is reduced centrally (hypothalamus) but not peripherally in *IL-6Ra KD* mice.

### 3.2 The adiposity of IL-6Ra KD mice is dependent on diet

**Figure 2:**
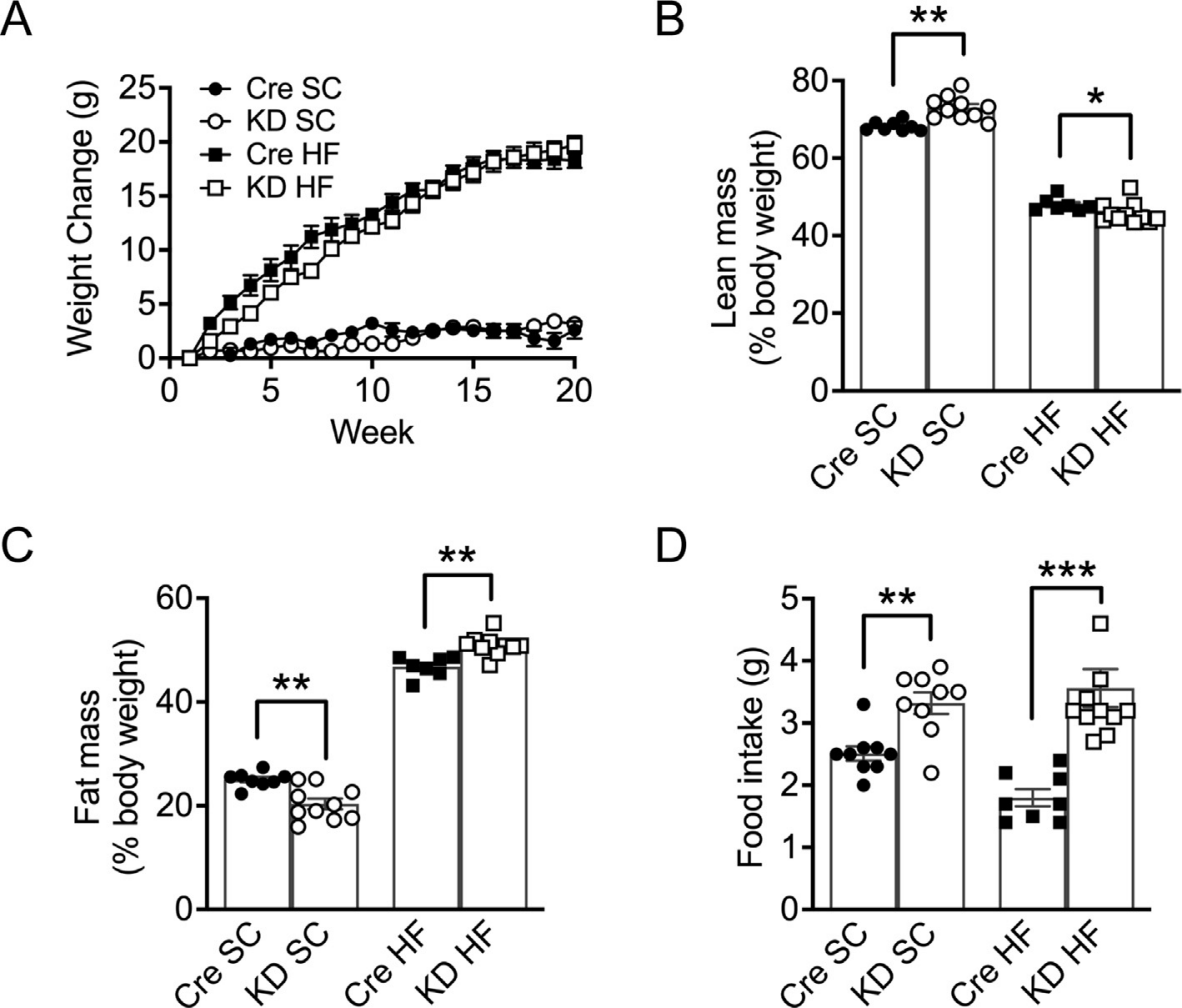
**Adiposity of*****IL-6Ra KD*****mice is dependent on diet.** A. Weight curves, showing the increase in body weight with time, of *Cre*^*+/-*^ and *IL-6Ra KD* mice on standard chow (SC) or high fat (HF) diet (*n* = 7–11/group). B. Lean mass (% body weight) of *IL-6Ra KD* and *Cre*^*+/-*^ mice on SC or HF diet, measured at week 20 (*n* = 7–11/group). C. Fat mass (% body weight) of *IL-6Ra KD* and *Cre*^*+/-*^ mice on SC or HF diet, measured at week 20 (*n* = 7–10/group). D. Daily food intake under *ad libitum* conditions, measured at week 9 in *IL-6Ra KD* and *Cre*^*+/-*^ mice on SC or HF diet (*n* = 7–11/group). Results represent mean values ± SEM. Data were analysed by two-way ANOVA followed by Tukey's post-hoc test (D) or Kruskal–Wallis followed by Dunn's post hoc test (B, C). ∗*P* < 0.05, ∗∗*P* < 0.01, ∗∗∗*P* < 0.001.

**Figure 3:**
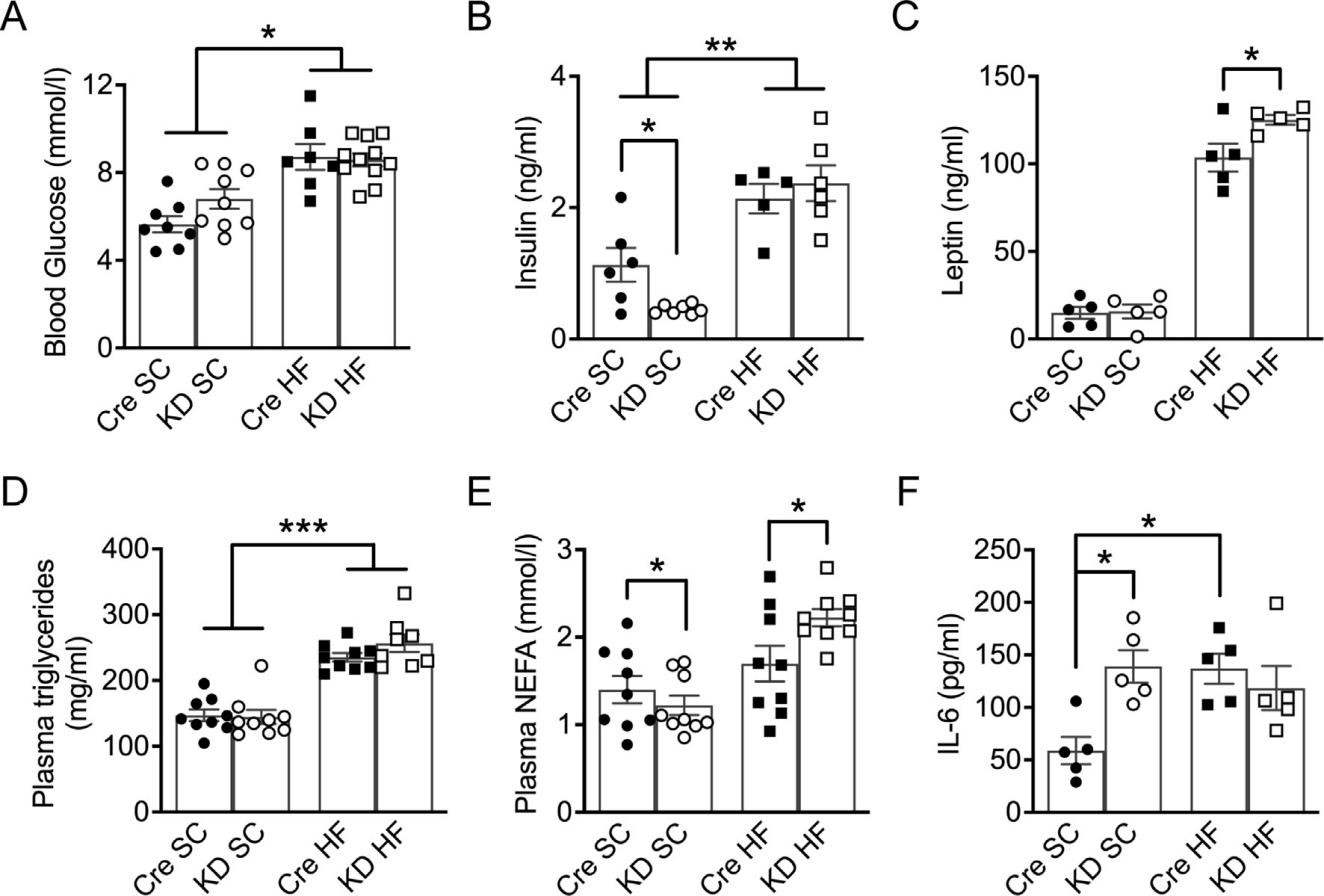
**IL-6Ra KD mice display altered plasma hormone and fat levels.** A. Fasted blood glucose levels in *IL-6Ra KD* and *Cre*^*+/-*^ mice measured at week 15 on SC and HF diet (*n* = 7–11/group). B. Fasted plasma insulin levels in *IL-6Ra KD* and *Cre*^*+/-*^ mice measured at week 19 on SC and HF diet (*n* = 5–7/group). C. Fasted plasma leptin levels in *IL-6Ra KD* and *Cre*^*+/-*^ mice measured at week 20 on SC and HF diet (*n* = 5/group). D. Fasted blood TG levels in *IL-6Ra KD* and *Cre*^*+/-*^ mice measured at week 20 on SC and HF diet (*n* = 8–9/group). E. Fasted blood NEFA levels in *IL-6Ra KD* and *Cre*^*+/-*^ mice measured at week 20 on SC and HF diet (*n* = 5/group). F. Fasted Blood IL-6 levels in *IL-6Ra KD* and *Cre*^*+/-*^ mice measured at week 20 on SC and HF diet (*n* = 5/group). Results represent mean values ± SEM. Data were analysed by two-way ANOVA followed by Tukey's post-hoc test. ∗*P* < 0.05, ∗∗*P* < 0.01, ∗∗∗*P* < 0.001.

*IL-6Ra KD* mice and *Cre*^*+/-*^ controls (12–14 weeks old) were continued on standard rodent chow (SC) diet for 20 weeks, and metabolic parameters were assessed. There was no significant effect of genotype on body weight gain (Figure 2A, *p* = 0.18), with both groups showing a positive weight trajectory throughout the study (effect of time, *p* < 0.001), with a similar amount of weight gained over the 20 weeks (weight change effect of genotype; *p* = 0.65). However, *IL-6Ra KD* mice on the SC diet were lighter than their Cre counterparts (Supplemental Fig. 1A) for the duration of the study (effect of genotype; *p* < 0.01; effect of time *p* < 0.01; genotype x time *p* < 0.01). As a fraction of body weight, *IL-6Ra KD* mice on an SC diet were slightly leaner (Figure 2B, *p* < 0.01) and had reduced body fat mass when compared to the *Cre*^*+/-*^ mice on the SC diet (Figure 2C, *p* < 0.01), although this difference was not significant on comparing the absolute weights (Supplemental Figs. 1B and C). The changes in body composition were not due to reduced food intake by *IL-6Ra KD* mice; rather, these mice demonstrated hyperphagia compared to *Cre*^*+/-*^ controls (Figure 2D, *p <* 0.01). Fasted blood glucose levels were comparable between *IL-6Ra KD* and *Cre*^*+/-*^ mice on SC diet (Figure 3A; *p* = 0.07). In contrast, fasted basal plasma insulin levels were lower in *IL-6Ra KD* mice on an SC diet compared to SC-fed *Cre*^*+/-*^ mice (Figure 3B, *p* < 0.05). There was no difference in plasma leptin (Figure 3C, *p* = 0.89) or plasma triglycerides (Figure 3D, *p* = 0.87) between genotypes on the SC diet. Circulating NEFA levels, however, were reduced (Figure 3E, *p <* 0.05) while plasma levels of IL-6 were elevated in *IL-6 Ra KD* animals when compared to *Cre*^*+/-*^ mice on SC diet (Figure 3F, *p* < 0.05)

As the effects of central IL-6 on food intake and glucose homeostasis are reported to be enhanced by obesity [14], we next examined the effect of 20 weeks HF diet on *IL-6Ra KD* and *Cre*^*+/-*^ mice (12–14 weeks old). HF feeding led to a significant increase in body weight of *IL-6Ra KD* and *Cre*^*+/-*^ mice throughout the study (Figure 2A, Supplemental Fig. 1A, effect of time *p* < 0.05), with no difference in the weight trajectory or final body weight change between genotypes (weight change; *p* = 0.39). In contrast to the SC-fed *IL-6Ra KD* mice, HF feeding resulted in a lower percentage lean mass (Figure 2B, *p* < 0.05), but not absolute lean mass (Supplemental Fig. 1B) and higher percentage body fat mass (Figure 2C, *p* < 0.01) and absolute fat mass (Supplemental Fig. 1C) of *IL-6Ra KD* mice, in comparison to HF-fed *Cre*^*+/-*^ mice. As observed for SC-fed mice, *IL-6Ra KD* mice exhibited a higher food intake than *Cre*^*+/-*^ controls on the HF diet (Figure 2D, *p* < 0.001). Fasting blood glucose was significantly higher in HF-fed animals compared to their SC-fed counterparts (effect of diet, *p* < 0.05), with no effect of genotype (Figure 3A, *p* = 0.95). Fasting insulin levels were raised by HF diet and were indistinguishable by genotype (Figure 3B, *p* = 0.52). The HF diet-mediated increase in fat mass was associated with raised plasma leptin, compared to SC-fed mice for both genotypes, with *IL-6Ra KD* mice exhibiting a higher plasma leptin level than *Cre*^*+/-*^ mice (Figure 3C, *p* < 0.05). Plasma triglyceride was increased by HF diet (effect of diet *p* < 0.001) irrespective of genotype (Figure 3D, *p* = 0.18). Circulating NEFA levels were higher in HF-fed *IL-6Ra KD* mice compared to HF-fed *Cre*^*+/-*^ controls (Figure 3E, *p* < 0.05). Plasma IL-6 levels (Figure 3F) were elevated following HF feeding in *Cre*^*+/-*^ controls; however, there was no effect of diet on IL-6 levels for *IL-6Ra KD* mice (*p* = 0.49).

### 3.3 IL-6Ra KD mice exhibit decreased activity and energy expenditure

**Figure 4:**
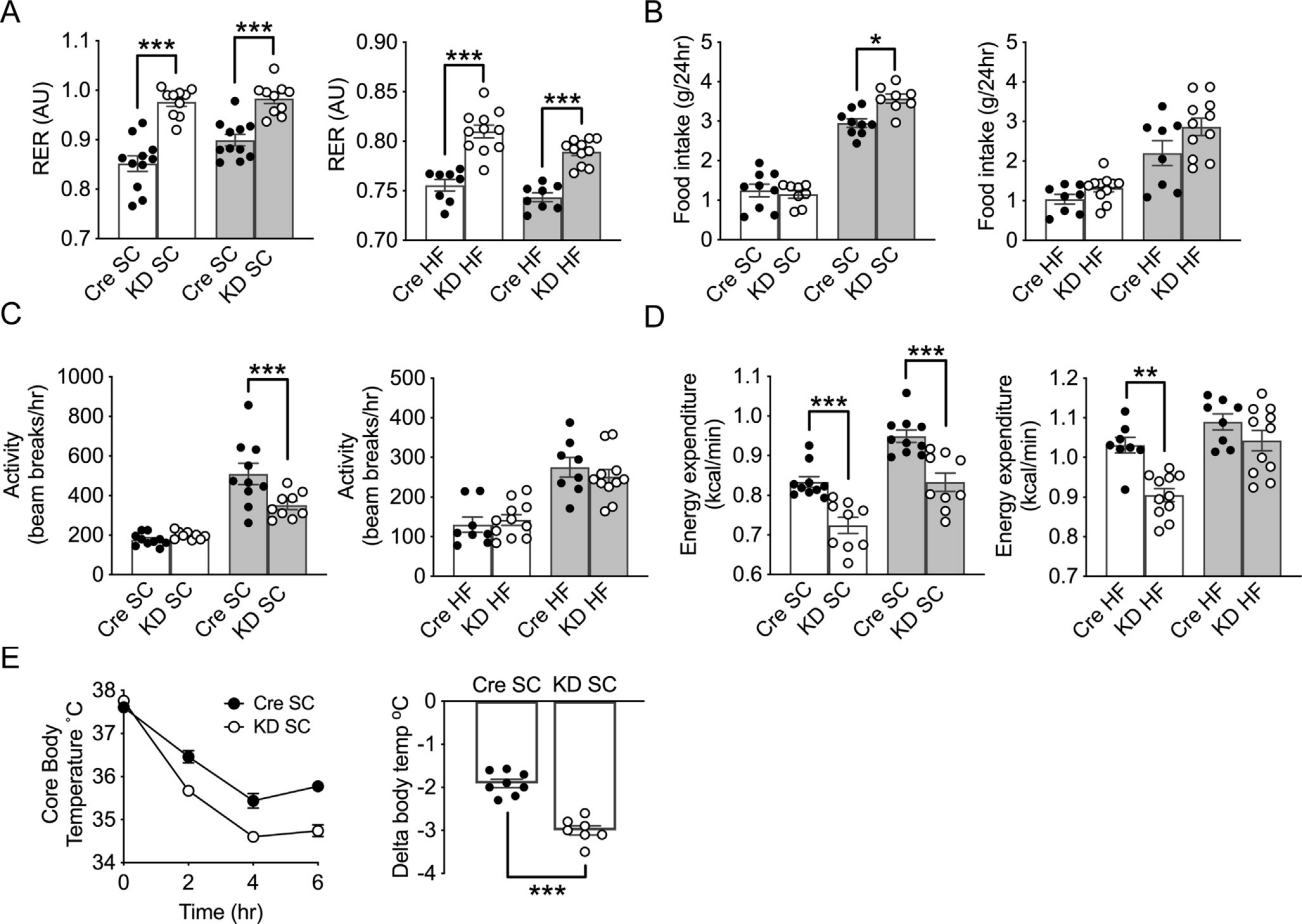
**Altered energy homeostasis in*****IL-6Ra KD*****mice.** A. Respiratory exchange ratio (RER), B. Food intake, C. Locomotor activity, and D. Energy expenditure, measured at week 20, for *IL-6Ra KD* and *Cre*^*+/-*^ mice during light (white bars) and dark (grey bars) cycles (*n* = 8–11/group). E. Core body temperature change with time in *IL-6Ra KD* and *Cre*^*+/-*^ mice on SC diet exposed to 4 °C. Change (delta) in body temperature at 6hrs. (*n* = 7–8/group). Results represent mean values ± SEM. Data were analysed by two-way ANOVA followed by Tukey's post-hoc test and unpaired Student's t-test (for E) ∗*P* < 0.05, ∗∗*P* < 0.01, ∗∗∗*P* < 0.001.

To investigate further the relationship between increased food intake and altered fat mass of *IL-6Ra KD* mice, indirect calorimetric measurements were made in SC- and HF-fed *IL-6Ra KD* and *Cre*^*+/-*^ mice. The respiratory exchange ratio (RER) was significantly higher for *IL-6Ra KD* mice in both light and dark phases on either diet (Figure 4A; *p* < 0.01), indicating that reduced central IL-6 signaling results in an increased preference for oxidation of glucose as a fuel source. HF-feeding resulted in a reduced RER for both genotypes, with *IL-6Ra* KD mice maintaining a relatively higher RER than *Cre*^*+/-*^ mice. These changes were accompanied by increased food intake during the dark phase (Figure 4B, *p* < 0.05) for SC-fed mice and a trend toward an increase in the dark phase for HF-fed *IL-6Ra KD* mice (*p* = 0.056). SC-fed, *IL-6Ra KD* mice were significantly less active during the dark phase than *Cre*^*+/-*^ mice (Figure 4C, *p* < 0.001). As anticipated, activity was significantly lower in HF-fed mice when compared to their SC-fed counterparts (effect of diet *p* < 0.05); however, there was no effect of genotype on activity.

*IL-6Ra KD* mice displayed a lower energy expenditure in comparison to *Cre*^*+/-*^ mice, with SC-fed *IL-6Ra KD* mice exhibiting reduced energy expenditure in both light and dark phases (Figure 4D, *p* < 0.001). In contrast, lower energy expenditure was only observed in the light phase for *IL-6Ra KD* mice on the HF diet (Figure 4D, *p* < 0.01). Additionally, when mean energy expenditure per mouse is plotted against lean body mass (Supplemental Figs. 2A and B) the data for SC- and HF-fed *IL-6Ra KD* and *Cre*^*+/-*^ mice lie on separate lines indicating that the reduced energy expenditure of *IL-6Ra KD* mice is an effect of genotype independent of mass. Mishra et al. [7] reported that IL-6 delivered centrally (to the lateral parabrachial nucleus; IPBN) in mice increases hyperthermia, whereas diminished IPBN IL-6 levels result in lower core temperature and reduced BAT thermogenesis. SC-fed *IL-6Ra KD* mice are characterised by lower energy expenditure and activity, higher RER and reduced plasma NEFA and fat mass, indicating that they may be less metabolically flexible. Therefore, we surmised that SC-fed *IL-6Ra KD* mice might be less able to cope with a cold challenge due to a reduced ability to utilise fat as an alternative fuel source when required. Thus, *IL-6Ra KD* and *Cre*^*+/-*^ mice were placed at 4 °C for 6 h, and core body temperature was measured by rectal probe at regular intervals. Core body temperature decreased over time for both genotypes (Figure 4E, effect of time *p* < 0.001); however, *IL-6Ra KD* mice were less able to maintain core body temperature, and the experiment was terminated after 6 h (effect of genotype *p* < 0.05). The reduction in body temperature (delta) throughout the study was significantly greater for *IL-6Ra KD* mice (Figure 4F, *p* < 0.001).

### 3.4 Increased steatosis in HFD-fed IL-6Ra KD mice

**Figure 5:**
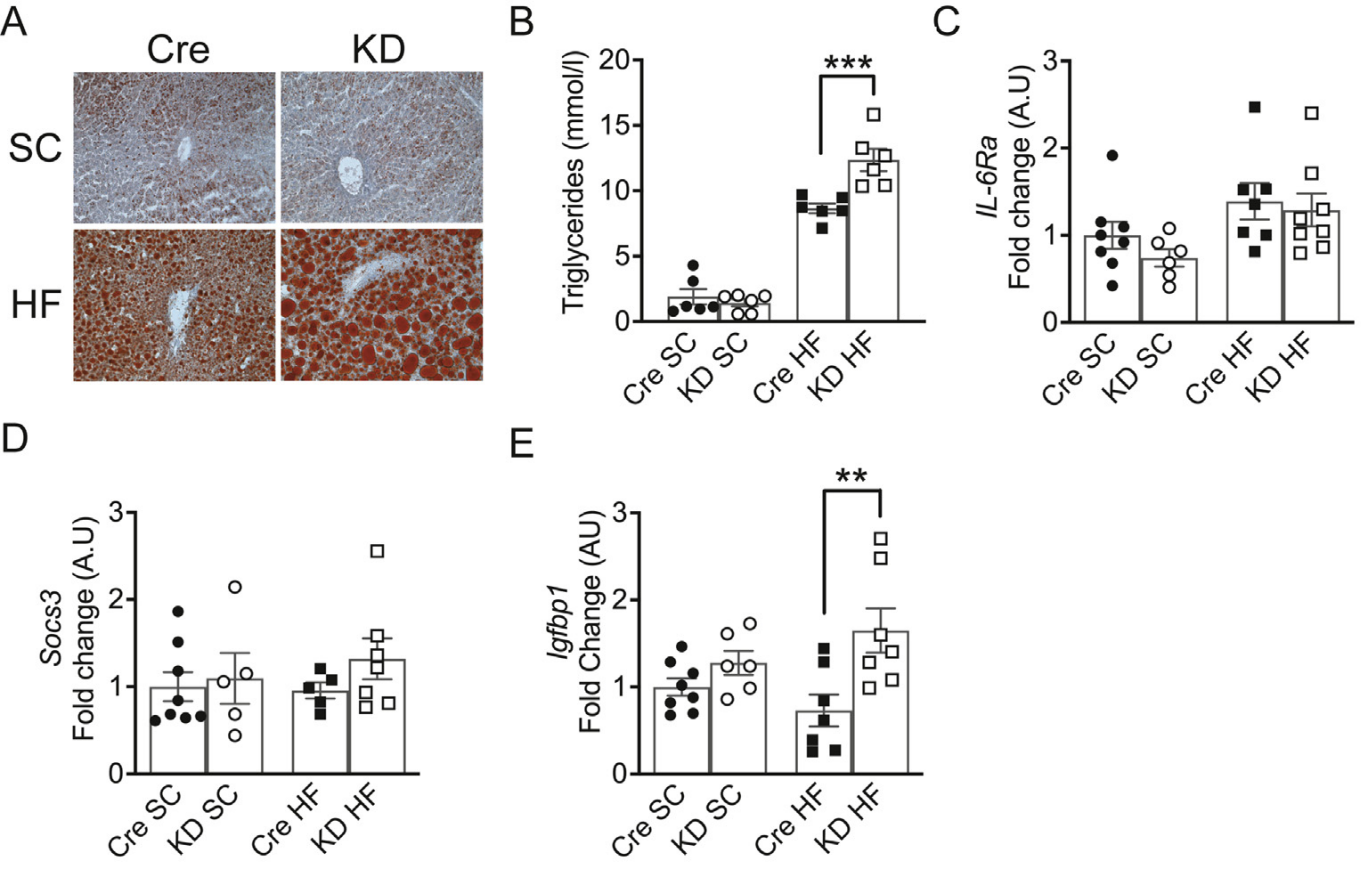
**HF-fed*****IL-6Ra KD*****mice display increased liver steatosis.** A. Oil Red-O staining of frozen sections of liver to detect lipid droplets (red) from SC- and HF-fed *Cre*^*+/-*^ and *IL-6Ra KD* mice. Images are representative of observations made on 6 mice per group. B. Liver triglyceride content in SC- and HF-fed *Cre*^*+/-*^ and *IL-6Ra KD* mice after 20 weeks diet (*n* = 6/group). Quantification of *IL-6Ra* (C), *Socs3* (D) and *Igfbp1* (E) expression in the liver of SC- and HF-fed *IL-6Ra KD* and *Cre*^*+/-*^ control mice (*n* = 5–8/group). Results represent mean values ± SEM. Data were analysed by two-way ANOVA followed by Tukey's post-hoc test (B) or Kruskal–Wallis followed by Dunn's post hoc test (C–E). ∗∗*P* < 0.01, ∗∗∗*P* < 0.001.

Obesity-driven increased plasma IL-6 in humans is reported to be associated with aggravated liver steatosis and fibrosis [34,35]. However, IL-6 has been reported to have beneficial effects on various mouse models of liver steatosis [36,37]. Indeed, lack of IL-6, or reduced IL-6 levels by neutralising antibody, have been shown to increase lipid accumulation and exacerbate HF diet-induced steatosis in rodents [12,18,38]. In contrast, chronic IL-6 administration has been reported to aggravate steatosis in diet-induced obese mice and in *IL-6*^*−/−*^ mice fed HF diet [39,40]. Thus, our understanding of how whole-body IL-6 signaling affects liver fat metabolism is incomplete. As *IL-6Ra KD* mice exhibited a small increase in whole-body fat mass, decreased EE and metabolic flexibility coupled with a relative increase in energy intake on HF-feeding, we speculated that energy storage in these mice may be associated with altered nutrient partitioning. Consequently, we examined lipid accumulation in livers of SC- and HF-fed *IL-6Ra KD* and *Cre*^*+/-*^ mice. Oil-red O staining of liver sections (Figure 5A) and liver triglyceride content (Figure 5B) from SC-fed mice reveal no significant difference in hepatic lipid load. As expected, mice HF-fed for 20 weeks displayed accumulation and expansion of lipid droplets in hepatocytes (Figure 5A) and increased triglyceride content (Figure 5B) for both genotypes, which was enhanced in *IL-6Ra KD* mice (*p* < 0.01 vs *Cre*^*+/-*^ HF, *p* < 0.001 vs KD SC). There was no detectable difference in liver *IL-6Ra* expression from *IL-6Ra KD* and *Cre*^*+/-*^ mice on either diet (Figure 5C), likewise transcript abundance of *Socs3*, liver levels of which have been suggested to play an important role in steatosis [[41], [42], [43]], was unaltered by either diet or genotype (Figure 5D). In addition, HF-fed *IL-6Ra KD* mice exhibited an increase in *Igfbp1* (Figure 5E), a predictive marker for liver lipid accumulation [44].

### 3.5 Impaired glucose tolerance in IL-6Ra KD mice

**Figure 6:**
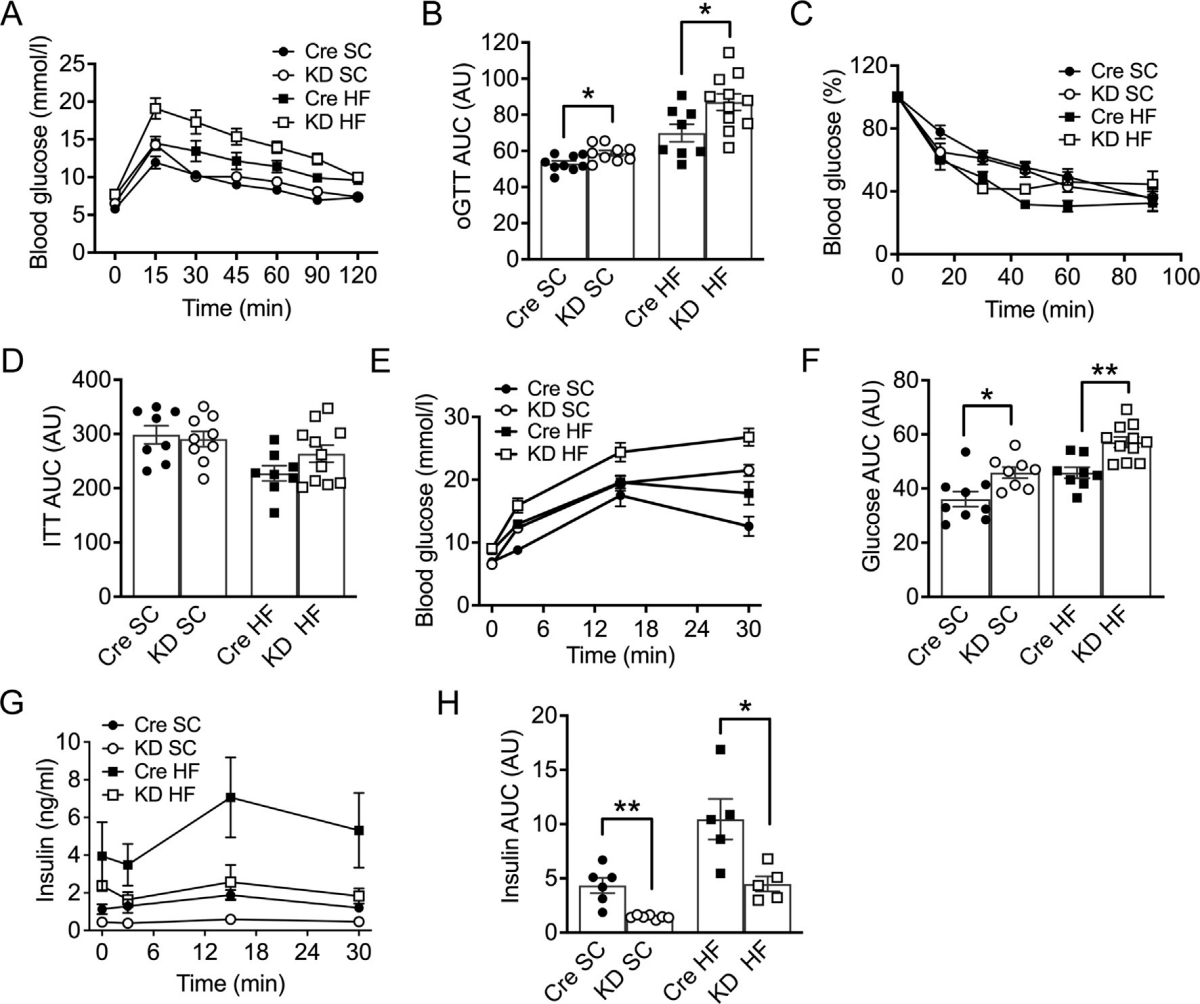
**Blunted glucose homeostasis and insulin secretion in*****IL-6Ra KD*****mice.** A. Oral glucose tolerance test in *IL-6Ra KD* and *Cre*^*+/-*^ mice, measured at week 10, on SC and HF diets (*n* = 8–11/group). B. Quantification of the area under the curve (AUC) for the total glycemic excursions shown in A. C. Insulin tolerance test (ITT) in *IL-6Ra KD* and *Cre*^*+/-*^ mice, measured at week 15, on SC and HF diets (*n* = 8–11/group). D. AUC for the total glycemic excursions shown in C. E. Blood glucose levels with time during oral glucose-stimulated insulin secretion (oGSIS) test in *IL-6Ra KD* and *Cre*^*+/-*^ mice, measured at week 19, on SC and HF diets (*n* = 8–11/group). F. AUC for the total glycemic excursions shown in E. G. *IL-6Ra KD* mice show impaired insulin secretion *in vivo* relative to *Cre*^*+/-*^ controls on SC and HF diets (*n* = 5–7/group). H. AUC for the total glycemic excursions shown in G. Results represent mean values ± SEM. Data were analysed by two-way ANOVA followed by Tukey's post-hoc test. ∗*P* < 0.05, ∗∗*P* < 0.01.

Central injection of IL-6 is reported to improve insulin sensitivity and glucose homeostasis, with a more pronounced action on obese mice [14]. Thus, we next examined the impact of reduced central IL-6 signaling in I*L-6Ra KD* mice on glucose disposal and insulin sensitivity. SC fed *IL-6Ra KD* and *Cre*^*+/-*^ mice were challenged with an oral glucose load and tolerance determined (oGTT). Glucose clearance was mildly impaired in SC *IL-6Ra KD* mice in comparison with *Cre*^*+/-*^ controls (Figure 6A, genotype x time, *p* < 0.05) with the AUC significantly increased in I*L-6Ra KD* mice (Figure 6B, *p* < 0.05). However, as shown in the ITT and derived AUC, insulin sensitivity was unaffected in the SC-fed mice (Figure 6C,D). As SC-fed *IL-6Ra KD* mice displayed reduced plasma insulin levels, we next investigated whether the difference in glucose disposal was due to a pancreatic defect. Consequently, SC-fed *IL-6Ra KD* and *Cre*^*+/-*^ mice underwent a glucose-stimulated insulin secretion test at week 19 of diet, which showed that *IL-6Ra KD* mice had impaired glucose clearance (Figure 6E, effect of time p < 0.01, effect of genotype p < 0.05 and genotype x time *p* < 0.01) and AUC (Figure 6F, *p* < 0.05), corroborating the oGTT findings at the 10-week SC feeding time point. Notably, the impaired glucose disposal was accompanied by a substantial suppression of insulin secretion in SC-fed *IL-6Ra KD* mice in comparison to *Cre*^*+/-*^ controls (Figure 6G, genotype x time *p* < 0.01) illustrated by the suppressed AUC for insulin (Figure 6H, *p* < 0.01).

To determine whether the increased fat mass of HF-fed *IL-6Ra KD* mice further impacted on glucose and insulin handling, obese *IL-6Ra KD* and *Cre*^*+/-*^ mice were also subjected to glucose and insulin tolerance tests. Glucose clearance was impaired in *IL-6Ra KD* mice (Figure 6A; genotype x time *p* < 0.01), with the AUC increased in *IL-6Ra KD* mice (Figure 6B, *p* < 0.05). In contrast, there was no difference in insulin sensitivity (Figure 6C; genotype *p* = 0.09) and the AUC (Figure 6D). As shown for SC-fed *IL-6Ra KD* mice, HF-fed *IL-6Ra KD* mice that underwent a glucose-stimulated insulin secretion test at week 19 of diet also displayed impaired glucose clearance (Figure 6E, effect of time p < 0.01, effect of genotype p < 0.05 genotype x time p < 0.01) as shown by an increased AUC (Figure 6F, *p* < 0.01). This impaired glucose disposal was also associated with a suppression of oral glucose-stimulated insulin secretion (Figure 6G, genotype x time *p* < 0.05) and a reduced AUC for insulin (Figure 6H, *p* < 0.05).

### 3.6 Ex vivo islets from IL-6Ra KD mice exhibit a normal pancreatic phenotype and GSIS

**Figure 7:**
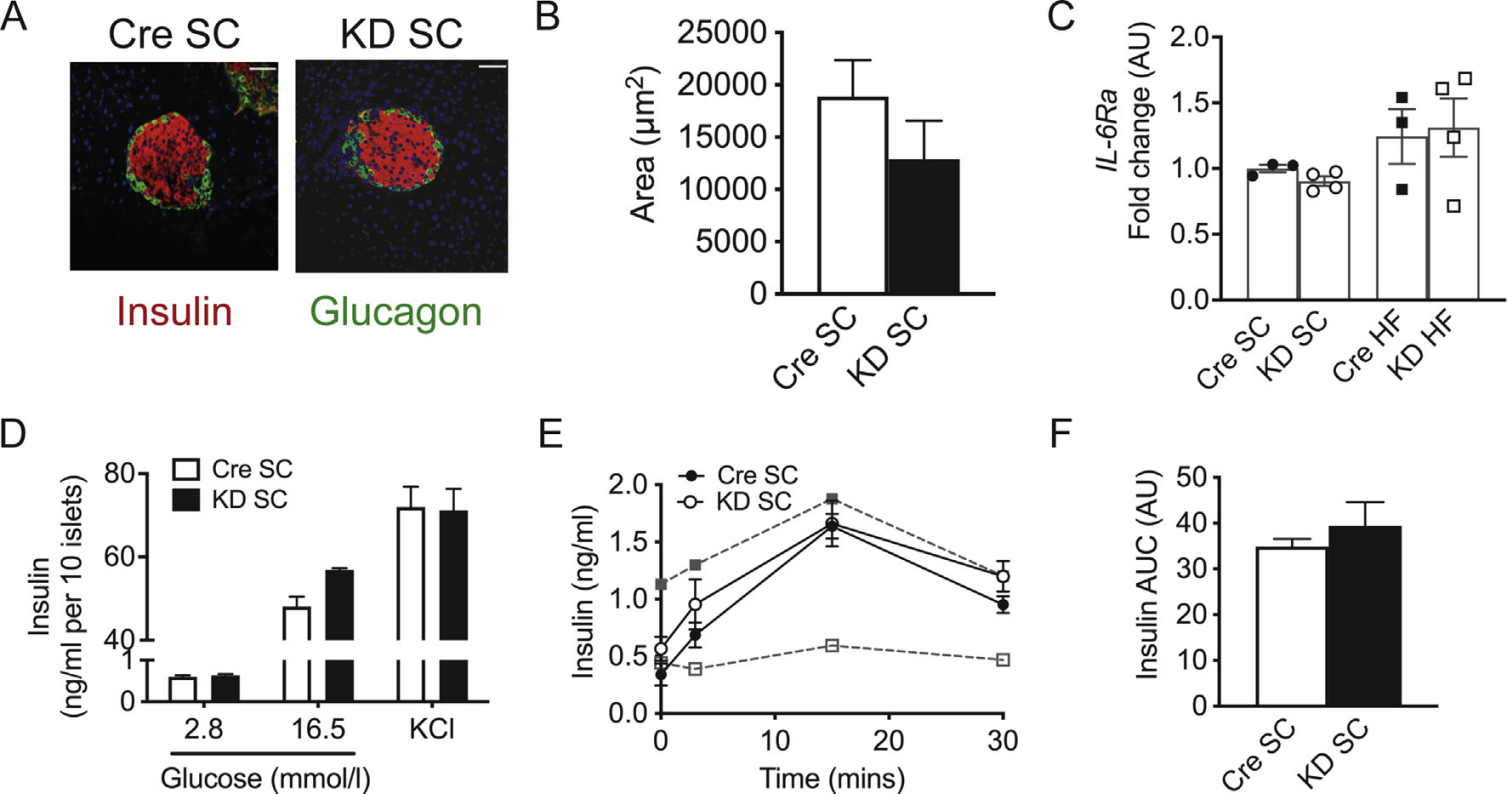
**Suppression of insulin secretion in*****IL-6Ra KD*****mice is centrally mediated.** A. Pancreatic sections from 8-week-old SC fed *IL-6Ra KD* and *Cre*^*+/-*^ mice, co-stained for insulin (red), glucagon (green) and DAPI (4′6′-diamidino-2-phenylindole; blue). B. Mean islet area of SC fed *Cre*^*+/-*^ (*n* = 20) and *IL-6Ra KD* (*n* = 26) mice. C. Islet *IL-6Ra* expression of *Cre*^*+/-*^ and *IL-6Ra KD* mice on SC and HF diet (*n* = 3–4/group) D. Insulin secretion from isolated SC fed *Cre*^*+/-*^ and *IL-6Ra KD* mouse islets in static cultures in response to 2.8 and 16.5 mmol/l glucose or following exposure to 30 mmol/l KCl (*n* = 3–5/group). E. OGSIS in SC-fed *Cre*^*+/-*^ and *IL-6Ra KD* mice 15 min after hexamethonium injection (30 mg/kg i.p.). The oGSIS data for SC-fed *Cre*^*+/-*^ (open squares) and *IL-6Ra KD* mice (closed squares) from Figure 6E are shown (grey symbols and broken line) for comparison. F. AUC for the insulin excursion for hexamethonium-treated mice shown in E (*n* = 4/group). Results represent mean values ± SEM. Data were analysed by Kruskal–Wallis followed by Dunn's post hoc test (C), two-way ANOVA followed by Tukey's post-hoc test (D) and Student's t-test (F).

To investigate this disruption of *in vivo* glucose homeostasis and insulin secretion in *IL-6Ra KD* mice, we examined pancreatic islet morphology and *ex vivo* GSIS. Pancreatic islets from SC-fed *IL-6Ra KD* and *Cre*^*+/-*^ mice were isolated, and immunohistochemistry was performed to identify β− and α-cells and determine the islet area. Pancreatic morphology was comparable (Figure 7A) with no significant difference in islet area (Figure 7B; *p* = 0.24) between genotypes. Islet *IL-6Ra* expression was unaffected by genotype or diet (Figure 7C; *p* = 0.45). GSIS was determined on isolated islets incubated with 2.8 or 16.5 mmol/l glucose (with 30 mmol/l KCl as a positive control) for 30 min. Insulin (Figure 7D) was secreted in a concentration-dependent manner in response to the glucose challenge. Notably, there was no difference in the amount of insulin released by isolated islets from *IL-6Ra KD* or *Cre*^*+/-*^ mice to either the high glucose or 30 mmol/l KCl challenge. Furthermore, islets isolated from WT controls responded to glucose and KCl challenge to a similar extent as *Cre*^*+/-*^ mouse isolated islets (Supplemental Fig. 3), indicating relatively normal GSIS of the *Cre*^*+/-*^ controls. The apparent inability of *IL-6Ra KD* mice to modulate their secretion of insulin *in vivo* in response to a glucose challenge suggests aberrant autonomic innervation of the pancreas. To explore this possibility further, SC-fed *IL-6Ra KD* and *Cre*^*+/-*^ mice were injected with the non-depolarising autonomic ganglionic blocker hexamethonium 15 min before performing an oGSIS. Hexamethonium blockade of ganglionic transmission restored *in vivo* insulin secretion in response to a glucose challenge in *IL-6Ra KD* mice to a level indistinguishable from *Cre*^*+/-*^ controls (Figure 7E, genotype x time *p* = 0.29), and as observed for the AUC (Figure 7F, p = 0.46).

## 4 Discussion

IL-6 is secreted from a wide range of cells, and, owing to the presence of classical- (through IL-6Ra) and trans-signaling (via IL-6/IL-6Ra complex association with gp130) pathways can elicit responses in most cells. Thus, IL-6 has pleiotropic properties and contributes to inflammation with pro-and anti-inflammatory effects described and the regulation of glucose and lipid metabolism [45]. Indeed, plasma IL-6 levels are raised by obesity and exercise and are considered part of an adaptive response to maintain glucose and energy homeostasis. However, the exact role of IL-6 in metabolic control is complex and currently not well understood, with conflicting outcomes on glucose and lipid homeostasis reported in rodent studies. Previous studies have demonstrated an important role for IL-6 signaling in the central nervous system in the context of metabolic homeostasis [[11], [12], [13], [14]]. Consequently, we wanted to reduce IL-6Ra mediated central signaling to examine glucose and energy homeostasis in lean and obese mice. The Nestin *Cre* mouse strain was used to drive Cre recombinase expression to delete the IL-6Ra gene in the brain. However, this transgene exhibits background effects that impact the mouse phenotype. Indeed, relevant to the rationale for this study, Nestin Cre mice have been reported to show a metabolic phenotype with altered body weight, fat distribution and insulin sensitivity [26,27]. Thus, following the expectation that reduced brain IL-6Ra expression would induce a metabolic phenotype with modified fat and glucose homeostasis, we used the Nestin Cre mice (*Cre*^*+/-*^) as the control comparison group. Although primarily used to delete genes in neuronal tissue, the Nestin promotor has also been reported to elicit non-specific gene deletion resulting in Cre expression in peripheral tissues [28,46,47].

Furthermore, it is commonly noted for Cre-loxP recombination that inefficient gene deletion may occur. Thus, to determine the level and tissue distribution of IL-6Ra gene deletion in the progeny of Nestin *Cre*^*+/-*^ and *IL-6Ra*^*flx/flx*^ mice, we examined brain IL-6Ra mRNA expression and IL-6 signaling fidelity in the hypothalamus, liver, skeletal muscle and white adipose tissues of these mice. IL-6Ra mRNA was not uniformly reduced in the brains of IL-6Ra “knockout” mice, with differential reduction of transcript levels compared to *Cre*^*+/-*^ controls in hippocampal and hypothalamic areas but not in frontal cortex or cerebellum. Thus, the outcome of the recombination event did not produce a brain-specific knockout mouse but one characterised by a region-specific knockdown of the target gene (*IL-6Ra KD*), predominantly in limbic areas. As this study was focused on phenotypic outcomes associated with altered whole-body glucose and lipid metabolism, reduced expression in these brain areas was relevant. Functional analysis on *ex vivo* basal-medial hypothalamic slice wedges using p-STAT3 levels as a functional readout of cytokine signaling strength demonstrated that the *IL-6Ra KD* mouse was characterised by blunted IL-6 signaling compared to *Cre*^*+/-*^ controls. However, no reduction in hypothalamic leptin signaling fidelity via the p-STAT3 pathway was observed, suggesting conserved central responses to this adipokine in the *IL-6Ra KD* mouse. Additionally, no attenuated IL-6 signaling was detected in liver, skeletal muscle, or white adipose tissue, key peripheral tissues associated with glucose and lipid homeostasis. This study did not assess the contribution of trans-signaling to overall central IL-6 signaling and the phenotype described for IL-6Ra KD mice. Still, both classical and trans-signaling were likely reduced by the overall reduction in IL-6Ra in limbic regions. Indeed, central trans-signaling predominates in IL-6-mediated systemic control of energy and glucose homeostasis [14]. Consequently, the data presented indicate that partial reduction of IL-6Ra limbic expression, and therefore IL-6-mediated signaling, is sufficient to modify whole-body energy, glucose, and homeostasis.

*IL-6Ra KD* mice exhibit similar growth curves to *Cre*^*+/-*^ mice, whether on SC or HF diet. However, SC-fed *IL-6Ra KD* mice were generally slightly lighter, with reduced fat mass compared to *Cre*^*+/-*^ mice, whereas *IL-6Ra KD* mice exhibit a small increase in fat mass and plasma leptin levels on HF-feeding. This increase in overall fat mass is highlighted by the increased liver steatosis in HF-fed *IL-6Ra KD* mice, compared to HF-fed *Cre*^*+/-*^ mice. The increased liver triglyceride content is not associated with altered peripheral IL-6 signaling, as plasma IL-6 levels, liver IL-6Ra expression do not differ from HF-fed *Cre*^*+/-*^ controls, indicating that central IL-6 signaling mechanisms likely play a key role in maintaining hepatocyte lipid homeostasis when challenged by high fat feeding. Triglyceride levels in hepatocytes is predominantly a balance between plasma uptake of fatty acids, fatty acid oxidation and *de novo* lipogenesis for accrual, and secretion of TG-rich lipoproteins and fatty acid oxidation for elimination [48]. Thus, further analyses of liver lipid flux and metabolism is required to determine the mechanism for the increase in liver steatosis.

Under SC feeding, *IL-6Ra KD* mice display a relatively mild phenotype with respect to glucose homeostasis, exhibiting unchanged fasting blood glucose and mildly impaired glucose disposal during a glucose tolerance test. This glucose intolerance is likely mediated by the reduced plasma insulin levels observed in the *IL-6Ra KD* mouse. Although SC-fed *IL-6Ra KD* mice exhibit elevated plasma IL-6 levels, they do not display insulin resistance, as denoted by the insulin tolerance tests. Thus, diminished central IL-6 signaling appears to have minimal impact on peripheral insulin sensitivity in *IL-6Ra KD* mice. Although plasma insulin is lower in the SC-fed *IL-6Ra KD* animals compared to *Cre*^*+/-*^ controls, this is not observed following HF-feeding, where both genotypes exhibit hyperinsulinemia. There was no change in SC-fed *IL-6Ra KD* islet size, morphology, or IL-6Ra transcript levels compared to SC-fed *Cre*^*+/-*^ mice with Nestin expression reported not to occur in islet endocrine cells [46]. Consequently, the decrease in plasma insulin observed in the *IL-6Ra KD* animals suggests that attenuated central IL-6 signaling reduces basal insulin secretion and that HF-feeding reduces the influence of this central drive. Previous studies on obese rats have indicated that high-fat feeding increases insulin secretion, in part by reduced sympathetic tone [49,50]. Furthermore, the suppression of *in vivo* glucose-stimulated insulin secretion in the SC- and HF-fed *IL-6Ra KD* animals, with no change in glucose-stimulated insulin secretion in *ex vivo* islets is also strongly supportive of an important role for IL-6Ra in the neural control of insulin secretion in response to a rise in plasma glucose. Indeed, the *ex vivo* islet data demonstrate that the pancreatic beta cells from *IL-6Ra KD* mice respond to raised extracellular glucose levels by secreting insulin in a manner identical to that of the *Cre*^*+/-*^ controls, indicating normal autonomous glucose sensitivity and transduction of metabolic signals. Autonomic control of insulin secretion involves a balance between the inhibitory effects of sympathetic nerve activity and the stimulatory actions of the parasympathetic arm [51]. The suppression of *in vivo* GSIS observed in the *IL-6Ra KD* animals suggested an altered autonomic drive to the pancreas resulting in enhanced inhibition of insulin secretion. This was tested by inhibiting autonomic ganglionic transmission through antagonising nicotinic acetylcholine receptor activity with the non-depolarising blocker hexamethonium. In hexamethonium-treated mice, the suppression of GSIS observed in *IL-6Ra KD* mice, compared to *Cre*^*+/-*^ controls, was prevented, and both genotypes displayed identical insulin release profiles. This central mechanism may work in conjunction with peripheral actions of elevated IL-6, which has been reported to promote insulin secretion indirectly by enhancing glucagon-like peptide (GLP)-1 production [52].

Although we cannot infer this outcome is specifically the result of a disruption to either the parasympathetic innervation of the beta-cell (with reduced output from the parasympathetic arm causing inhibition of insulin secretion) or the sympathetic innervation (with increased sympathetic output inhibiting insulin secretion). On balance, we think the latter is more likely as (i) the sympathetic nervous system is considered to exert tonic inhibition of the endocrine pancreas and is expected to influence basal and glucose-stimulated insulin secretion and (ii) parasympathetic control of islet function is thought to be more involved in the potentiation of insulin secretion during a hyperglycemic excursion. In addition, hexamethonium will have the effect of preventing all autonomic transmission from sympathetic and parasympathetic nerve terminals i.e. not exclusively affecting norepinephrine and/or acetylcholine. Consequently, although central IL-6 signaling appears to play an important role in the insulin secretion response to raised plasma glucose potentially by acting as a brake on the tonic sympathetic output to pancreatic beta cells further studies are required to substantiate this hypothesis. Of note, plasma glucagon levels were also suppressed in response to an oral glucose load in IL-6Ra animals (data not shown), analogous to the blunted glucagon secretion in response to LPS reported in IL-6-KO mice [53].

*IL-6Ra KD* mice exhibit hyperphagia, compared to *Cre*^*+/-*^ controls, independent of diet, in agreement with previous studies showing that central IL-6 signaling suppresses feeding [11,13,14]. Increased central IL-6 signaling has been reported to increase energy expenditure/oxygen consumption, raise body temperature and lower locomotor activity [11,13,54]. Thus, our finding that *IL-6Ra KD* mice also exhibit reduced energy expenditure and locomotor activity emphasises the importance of central IL-6 signaling in maintaining energy homeostasis. The marked increase in RER observed in *IL-6Ra KD* mice, whether on SC and HF diet, indicates a shift towards an increased share of energy from glucose oxidation at the expense of fat oxidation and so contributes to reduced energy expenditure. Although central IL-6 is reported to have no impact on RER in rats [12,54], IL-6 deficient mice have been reported to exhibit increased RER [[55], [56], [57]]. Thus, reduced central IL-6 signaling is likely associated with decreased fat utilisation and metabolic inflexibility in switching fuels under metabolic stress such as HF-feeding. The inability of *IL-6Ra KD* mice to maintain core body temperature under cold challenge agrees with previous studies on cold exposure of IL-6 deficient and central *IL-6Ra* conditional knockdown mice [56,58], indicating diminished central IL-6 signaling is also associated with a reduced ability to switch fuels for thermogenesis and maintain energy homeostasis. Additional studies are required to delineate the exact molecular basis and neural pathways in hypothalamic, IPBN and hindbrain regions [7,14,20,21] for these changes in whole-body energy homeostasis. Studies have indicated that peripheral IL-6 blockade with tocilizumab may improve insulin sensitivity and glycemic control in humans with and without diabetes, where plasma IL-6 is elevated [[59], [60], [61]]. However, tocilizumab displays low brain penetrance, and thus, it is presently unclear whether drugs that also target central IL-6 signaling in humans will show beneficial metabolic outcomes.

## 5 Conclusion

Central IL-6Ra signaling is associated with glucose homeostatic control mechanisms, contributing to the regulation of insulin secretion, food intake, lipid deposition and energy expenditure. A potential mechanism linking central IL-6Ra signaling efficacy and control of metabolic homeostasis is the modulation of autonomic output activity to peripheral tissues. This is supported by the finding that blockade of ganglionic transmission recovers *in vivo* GSIS in the *IL-6Ra KD* mouse. Consequently, raised plasma IL-6 levels and increased central IL-6 signaling likely play an essential role as part of an adaptive mechanism to metabolic stress (e.g., HF-feeding, cold-exposure, exercise) through altered ANS output by contributing to control of insulin secretion and fuel usage flexibility.

## Authors’ contributions

**A.D.M.:** Conceptualization, Methodology, Formal analysis, Investigation, Writing – Original draft, Writing - Review & Editing. **A.Y.**: Methodology, Formal analysis, Investigation. **J.R.G.:** Investigation. **J.M.R.T:** Investigation. **R.J.M.**: Conceptualization, Methodology, Writing – Review & Editing, Supervision, Funding acquisition. **M.L.J.A**.: Conceptualization, Methodology, Writing – Original draft, Writing - Review & Editing, Visualization, Supervision.
