# Supplementary material for: Central deficiency of IL-6Ra in mice impairs glucose-stimulated insulin secretion
Source: Mol Metab. 2022 Apr 22;61:101488. doi: 10.1016/j.molmet.2022.101488 (PMC9065900; doi:10.1016/j.molmet.2022.101488)
Supplement: Multimedia component 1 [file mmc1.pdf]

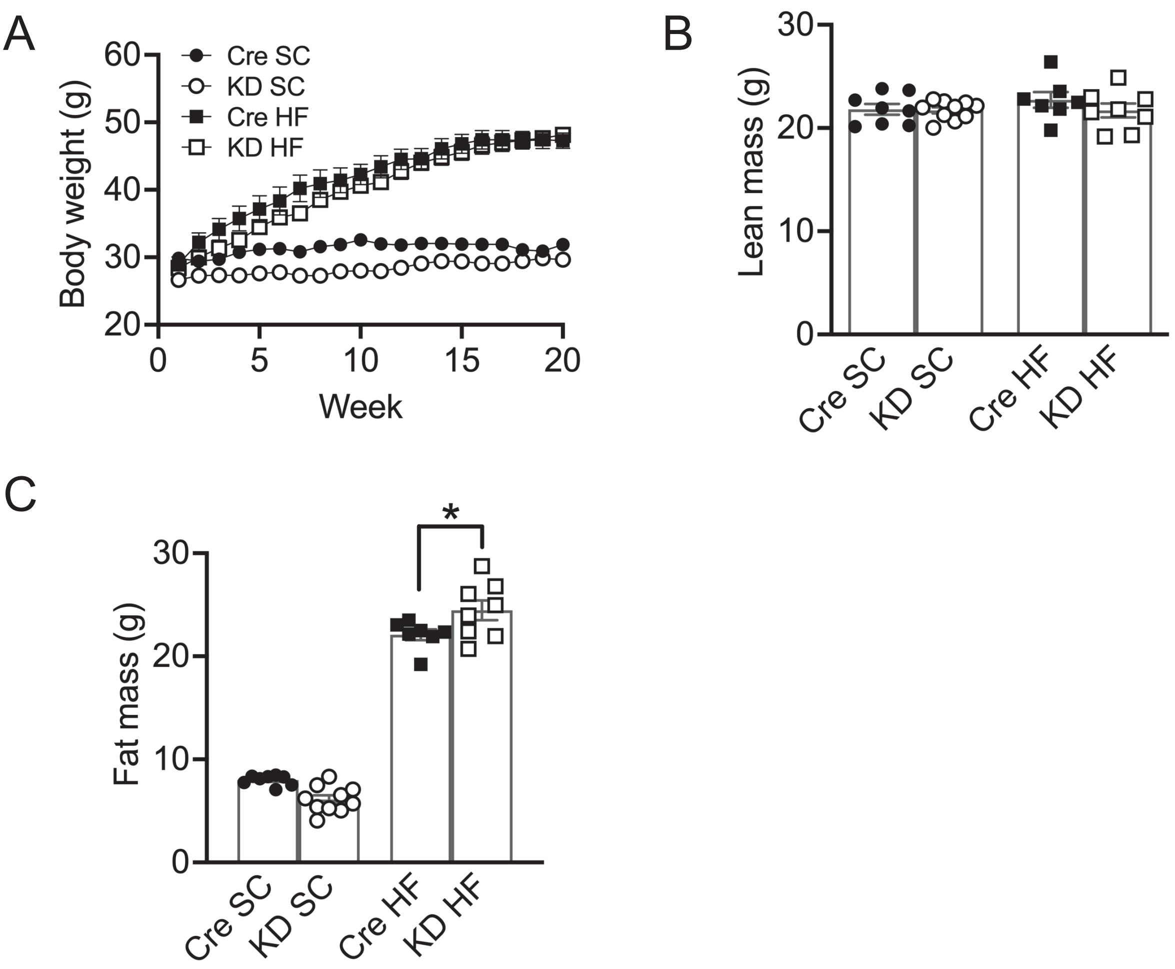

Supplemental Figure 1. Adiposity of *IL-6Ra KD* mice
A. Body weight curves of *Cre*$^{+/-}$ and I*L-6Ra KD* mice on SC and HF diet (*n* = 7-11/group). B. Lean mass of *Cre*$^{+/-}$ and *IL-6Ra KD* mice on SC and HF diet measured at week 20 (*n* = 7-11/group). C. Fat mass of *Cre*$^{+/-}$ and *IL-6Ra KD* mice on SC and HF diet, measured at week 20 (*n* = 7-10/group). Results represent mean values ± SEM. Data in B,C analysed by two-way ANOVA followed by Tukey's post-hoc test. * $P < 0.05$.
