# Supplementary material for: Central deficiency of IL-6Ra in mice impairs glucose-stimulated insulin secretion
Source: Mol Metab. 2022 Apr 22;61:101488. doi: 10.1016/j.molmet.2022.101488 (PMC9065900; doi:10.1016/j.molmet.2022.101488)
Supplement: Multimedia component 2 [file mmc2.pdf]

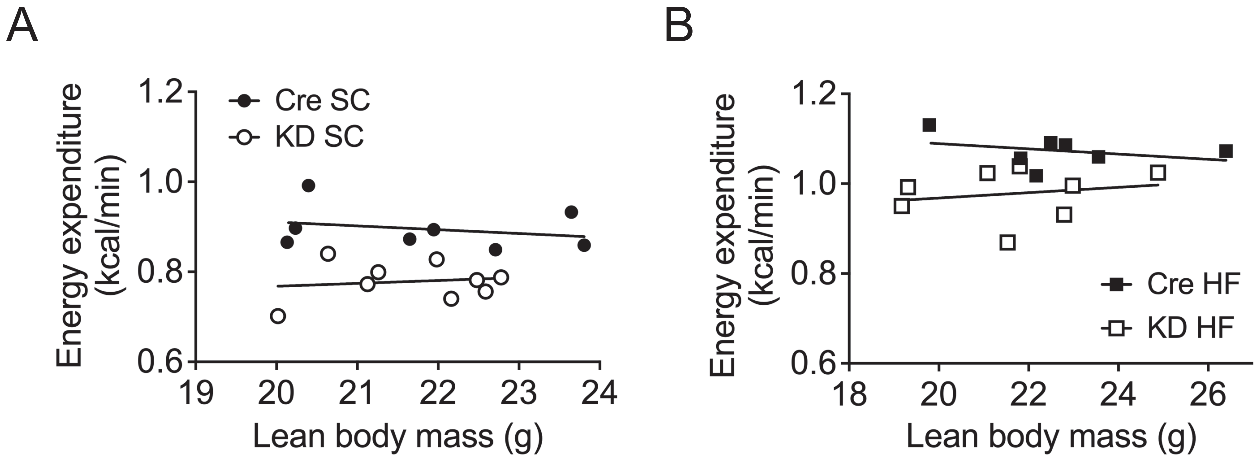


**Supplemental Figure 2. Individual mouse energy expenditure**

Energy expenditure (kcal/min) is plotted against lean body mass (g) for *Cre*$^{+/-}$ and *IL-6Ra KD* mice (*n* = 7-9/group) on (A) SC and (B) HF diet. Lines shown are fitted regression.
