# Supplementary material for: Central deficiency of IL-6Ra in mice impairs glucose-stimulated insulin secretion
Source: Mol Metab. 2022 Apr 22;61:101488. doi: 10.1016/j.molmet.2022.101488 (PMC9065900; doi:10.1016/j.molmet.2022.101488)
Supplement: Multimedia component 3 [file mmc3.pdf]

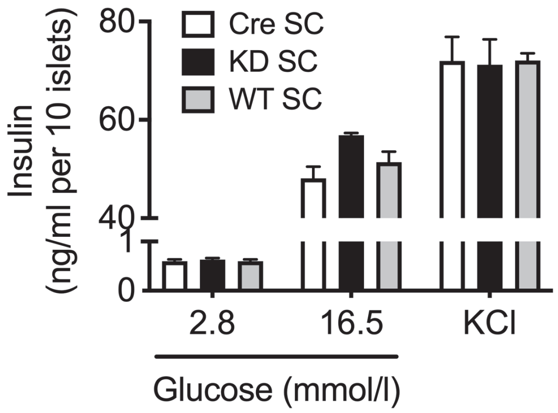

Supplemental Figure 3. Isolated islet insulin secretion
Insulin secretion from isolated SC fed *Cre*$^{+/-}$, I*L-6Ra KD* and *WT* mouse islets in static cultures in response to 2.8 and 16.5 mmol/l glucose or following exposure to 30 mmol/l KCl (*n* = 3-5/group).
